# Survival after Cardiac Arrest and Changing Task Profile of the Cardiac Arrest Team in a Tertiary Care Center

**DOI:** 10.1100/2012/294512

**Published:** 2012-04-30

**Authors:** Patrick Möhnle, Volker Huge, Jan Polasek, Isabella Weig, Rolf Atzinger, Uwe Kreimeier, Josef Briegel

**Affiliations:** Klinik für Anaesthesiologie der Universität München, Marchioninistraße 15, 81377 München, Germany

## Abstract

*Background*. The characteristics of in-hospital emergency response systems, survival rates, and variables associated with survival after in-hospital cardiac arrest vary significantly among medical centers worldwide. Aiming to optimize in-hospital emergency response, we performed an analysis of survival after in-hospital cardiopulmonary resuscitation and the task profile of our cardiac arrest team. *Methods*. In-hospital emergencies handled by the cardiac arrest team in the years 2004 to 2006 were analyzed retrospectively, and patient and event characteristics were tested for their associations with survival after cardiopulmonary resuscitation. The results were compared to a similar prior analysis for the years 1995 to 1997. *Results*. After cardiopulmonary resuscitation, the survival rate to discharge was 30.2% for the years 2004 to 2006 compared to 25.1% for the years 1995 to 1997 (difference not statistically significant). Survival after one year was 18.5 %. An increasing percentage of emergency calls not corresponding to medical emergencies other than cardiac arrest was observed. *Conclusions*. The observed survival rates are considerably high to published data. We suggest that for further improvement of in-hospital emergency response systems regular training of all hospital staff members in immediate life support is essential. Furthermore, future training of cardiac arrest team members must include basic emergency response to a variety of medical conditions besides cardiac arrest.

## 1. Background

The first reports of closed-chest cardiopulmonary resuscitation (CPR) for in-hospital cardiac arrest were published more than 60 years ago [1]. In the meantime, research on resuscitation has considerably increased; guidelines for cardiac resuscitation have been implemented on an international level and have undergone several substantial changes [2]. The institution of cardiac arrest teams for in-hospital resuscitation has become a standard for many medical centers worldwide. Still, the reported survival rates vary significantly with the center, patient, and event characteristics [3]. Over the last years, potential improvements of in-hospital medical emergency systems have increasingly become a matter of debate. Particularly, the concept of medical emergency teams providing in-hospital medical assistance early in deteriorating patients, aiming to lower the incidence of cardiac arrests, is under discussion [4, 5]. Aiming to optimize in-hospital medical emergency response, we sought to gain insight into the tasks and responsibilities of our established cardiac arrest team system. Our primary aim was to analyze survival after in-hospital cardiac arrest at our center. The secondary objective was to analyze the characteristics of medical emergencies treated by the cardiac arrest team.

## 2. Methods

### 2.1. Hospital Setting

The hospital is a 1400-bed university tertiary care center covering all medical and surgical disciplines. In-hospital medical emergency response is provided by a cardiac arrest team consisting of at least one specially trained anesthesiology resident on duty in an intensive care unit and at least two specially trained anesthesia nurses. The team is available 24 hours a day. Additionally, experienced senior anesthesiologists are on duty in the hospital and available at any time. Resuscitation training for the team members consists of regular institutional simulation-based ACLS (advanced cardiac life support) courses. Alarm calls can be placed by any person including hospital staff, visitors, and patients, from any phone in the hospital building and are dispatched via two-way radio to the team members who immediately hurry to the emergency site. There are no predefined criteria for emergency calls; all calls corresponding to any medical emergency situation as perceived by the caller are treated as emergency calls. Generally, the time from the emergency call to the arrival of the team is aimed to be below two minutes. The hospital main building consists of 16 floors with a total length of 200 m. Standardized emergency kits including airway devices, emergency drugs, oxygen, suction units, and defibrillators are placed in each ward throughout the building. For this retrospective analysis, the archived documentation of the cardiac arrest team and all available respective patients' charts for the complete years from 2004 to 2006 were reviewed and analyzed. The standard records of the cardiac arrest team consist of a concise standard paper documentation including patient identification, date, times, site, and all relevant medical parameters corresponding to those listed in Table 1. The results were compared to a prior similar study including the years 1995 to 1997.

### 2.2. Outcomes

Outcomes were defined as return of spontaneous circulation (ROSC), survival to 24 hours, survival to hospital discharge, and survival to 12 months. Return of spontaneous circulation was defined as the restoration of circulation for at least 20 minutes without CPR. The vital status after 12 months was confirmed either by patient chart or by telephone interview. For this retrospective study, ethical approval was waived by the IRB.

### 2.3. Analysis

Univariate logistic regression was applied to test the association of each evaluated parameter with each outcome. Variables with a statistically significant association (*P* < 0.05) were then further evaluated by stepwise backward multivariable logistic regression analysis (removal at *P* > 0.1). Statistical analyses were performed with MedCalc for Windows, version 11.3.3.0 (MedCalc Software, Mariakerke, Belgium).

## 3. Results

**Figure 1:**
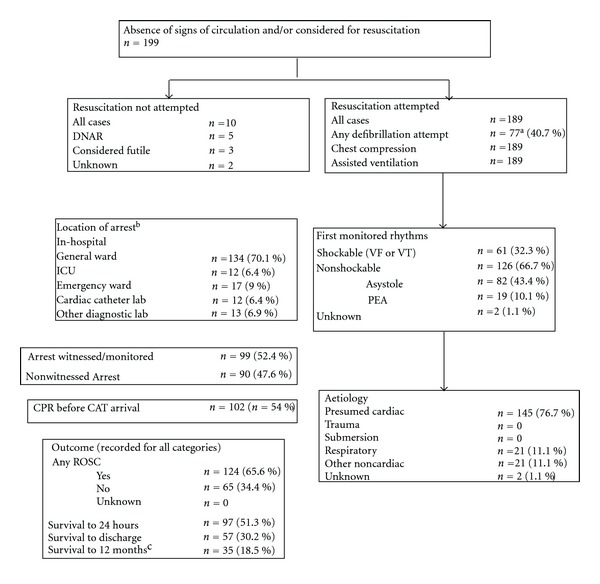
Utstein style chart for the core data. ^a^missing data on defibrillation attempts in one patient; ^b^missing data on the location of arrest in one patient; ^c^missing data on the vital status after 12 months in 7 patients.

In the three-year period from January 2004 to December 2006, the cardiac arrest team received 778 alarm calls. In 187 cases, the assistance of the emergency team was not needed upon arrival of the team due to false alarms or improved patient conditions. In 591 cases, the team provided assistance in medical emergency situations. In 199 patients, cardiac arrest was diagnosed, and in 189 patients, cardiopulmonary resuscitation was performed (Figure 1). In two of these patients, cardiopulmonary resuscitation was provided twice. The remaining 390 emergency calls, excluding cardiac arrests, were classified by the cardiac arrest team physician as airway/respiratory problems in 116 cases (29.7%), neurological emergencies in 75 cases (19.2%), syncopation/collapse in 68 cases (17.4%), cardiac emergencies other than cardiac arrest in 52 cases (13.3%), hemorrhage in 20 cases (5.1%), trauma in 14 cases (3.6%), and other/unclassified in 45 cases (11.5%).

### 3.1. Survival

After CPR, in 124 (65.6%) patients return of spontaneous circulation (ROSC) for at least 20 minutes was achieved. Univariate analyses revealed positive relations of ROSC with the variables recent history of surgery as wells as presumed cardiac etiology and negative associations with total dose of vasopressin, abnormal pupil findings, number of defibrillations, duration of CPR, age, and total dose of adrenaline.

Ninety-seven (51.3%) patients survived to 24 hours. In univariate comparison, survival to 24 hours was positively related to initial rhythm shockable, CPR before arrival of the cardiac arrest team, presumed cardiac etiology, witnessed arrest and was inversely related to total dose of vasopressin, female sex, duration of CPR, age, and total dose of adrenaline.

Fifty-seven (30.2%) patients could be discharged alive. In univariate analysis, improved survival was associated with the factors: CPR before arrival of the cardiac arrest team, recent history of surgery, presumed cardiac etiology, and witnessed arrest, whereas the variables total dose of vasopressin, initial rhythm shockable, abnormal pupil findings, duration of CPR, and total dose of adrenaline were negatively related to survival.

Finally, after 12 months, 35 (18.5%) patients were found to be alive, with missing data on the vital status of 7 patients. Univariate statistical analysis revealed positive associations of survival after 12 months with shockable initial rhythm, recent history of surgery, presumed cardiac etiology, witnessed arrest and negative associations with total dose of vasopressin, duration of CPR, age, and total dose of adrenaline.

**Figure 2:**
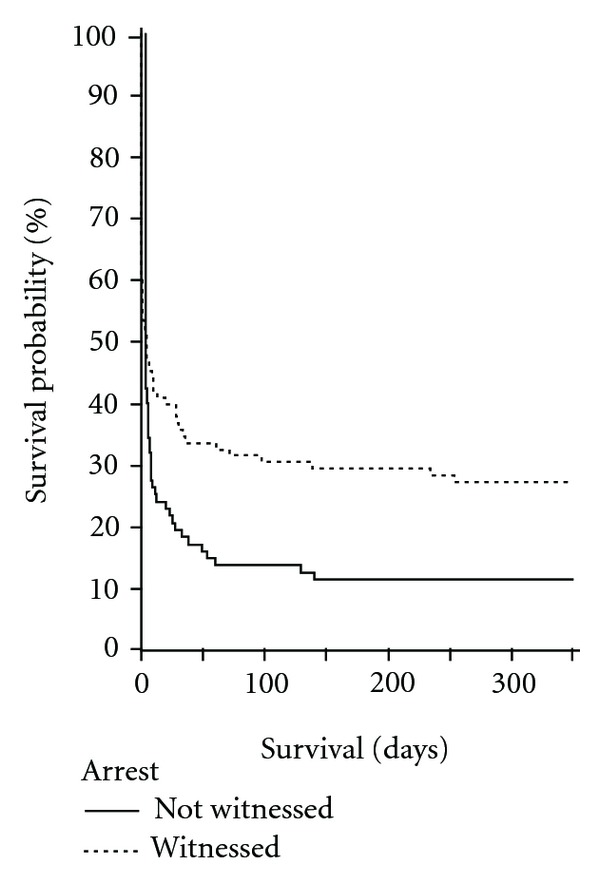
Kaplan-Meier survival curve for survival probability in relation to witnessed arrests (*P* = 0.001).

Results of the multivariate analyses for each point of time are depicted in Table 2. Survival after witnessed versus nonwitnessed arrest is displayed in Figure 2.

### 3.2. Patient Characteristics

In the 189 patients receiving CPR, the mean age was 65.2 (±16.1) years. The oldest patient was 95 years old, and the youngest patient was an infant of six weeks. One hundred and thirty-two (69.8%) patients were male, and 57 (30.2%) patients were female. Twenty-eight (14.8%) patients had undergone surgery during the respective hospitalization before the event. In 57 (30.2%) patients, a malignant disease had been diagnosed (Table 1).

In 145 (76.6%) patients, cardiac etiology was presumed for cardiac arrest. Presumed cardiac etiology was statistically not associated with age; however, the percentage of presumed cardiac etiology was higher in male patients compared to female patients (82.3% versus 66.7%, *P* = 0.03).

Coronary artery disease had been noted as a medical diagnosis in the patient chart for the respective hospitalization for 62 (32.8%) patients. Further medical diagnoses in the patient charts were congestive heart failure in 20 (10.6%) patients, renal insufficiency in 17 (9.0%) patients, diabetes in 13 (6.9%) patients, COPD in 12 (6.3%) patients, cerebrovascular disease in 8 (4.2%) patients, pneumonia in 4 (2.1%) patients, and sepsis in 3 (1.6%) patients.

### 3.3. Event Characteristics

In 134 (70.1%) cases, the location of cardiac arrest was a general ward. Twelve patients suffered from cardiac arrest in an intensive unit or an intermediate care unit. In 17 patients, the event took place in the emergency ward, and in 12 patients, cardiac arrest was diagnosed in the cardiac catheterization lab. There was no preference for the occurrence of cardiac arrest with respect to time of day (daytime defined as 8:00 AM to 7:59 PM, *n* = 96; night time, *n* = 89; *P* = 0.65). The mean duration of CPR was 20.4 min (SD 17.7 min; median duration 18.0 min, IQR 5.0/29.0). The shortest documented period of CPR was 1 minute, and the longest 100 minutes.

For CPR, adrenaline was used in 170 patients. Vasopressin was additionally used in 49 patients (Table 1). The first monitored rhythm was ventricular fibrillation or ventricular tachycardia and was therefore classified as shockable in 61 patients (32.3%). The pupil size upon arrival of the cardiac arrest team was recorded to be normal in 34 (18.0%) patients and dilated in 77 (40.7%) patients, with values missing for 78 (41.3%) patients. The pupillary reaction to light was described to be present in 6 patients (3.2%) and absent in 55 patients (29.1%), with missing values for 128 (67.8%) patients.

### 3.4. Survival after Cardiac Arrest from 1995–1997

Over the three-year period from January 2005 to December 1997, 607 in-hospital emergency alarms were reported (false alarms excluded). In 267 (44.0%) patients, cardiac arrest was diagnosed; these patients underwent CPR. The mean age was 63.8 (±16.4) years. Eighty percent of alarm dispatches originated from general wards. ROSC was achieved in 70.7% (189 patients); the survival rate after 24 hours was 45.7% (122 patients), and 67 patients (25.1%) survived to hospital discharge. For the difference in the survival rate to discharge compared to the time period from 2004 to 2006 (25.1% versus 30.2%), statistical significance could not be proven (*P* = 0.225). Neither patient age nor the time of day of the alarm was associated with survival. The final model of the logistic regression analysis for survival to discharge revealed the following three significant factors: initial ECG rhythm (sinus versus VF/VT: OR 3.0, 95% CI 0.98–8.56, *P* = 0.054; sinus versus asystolia: OR 2.3, 95% CI 0.87–6.31), witnessed arrest (OR 9.3, 95% CI 3.83–22.66; *P* < 0.001) and duration of CPR (≤15 min versus >15 min: OR 3.0, 95% CI 1.14–7.76; *P* = 0.026). With this model, 83.0% of all cases could be correctly classified as survival and non-survival.

## 4. Discussion

Survival to hospital discharge after in-hospital CPR has been described to vary from 0% to 42% in the literature, with the most common range between 15% and 20% [3]. We found survival rates in our center of 30.2% for the years from 2004 to 2006 and 25.1% for the years from 1995 to 1997. The observed rates are in an acceptable range compared to the data published in the literature and are higher compared to the most recently published reports on large patient numbers [6, 7]. For survival after 12 months, a rate of 18.5% was calculated under the assumption that seven patients, who could not be reached at followup, were deceased. The comparison of survival rates at discharge and at one-year followup revealed a high number of patients (23/57, 40.4%) who died or were lost to followup in the first year after in-hospital CPR, most probably reflecting serious underlying diseases. This rate is consistent with the literature, where a range between 53% and 86% for one-year survival for the originally discharged patients has been reported [3, 8–10].

Regarding the factors associated with survival to discharge, multivariate analyses for both time periods, 2004 to 2006 and 1995 to 1997, revealed the same set of variables: the first monitored cardiac rhythm and duration of CPR. These factors have been previously described for their association with survival. Ventricular fibrillation/ventricular tachycardia (VF/VT) as the first detected rhythm is known to impact survival [3, 8, 10–22], and the duration of CPR has frequently been shown to be associated with a worsened outcome [12–21, 23]. The analyses on the factors associated with survival furthermore revealed that presumed cardiac etiology was one important variable associated with improved long-term survival after 12 months and for short-term survival to 24 hours. Survival rates for patients with suspected myocardial infarction have been repeatedly reported to be elevated compared to those for patients with other conditions, most probably due to treatable arrhythmias as the underlying cause of arrest [7, 15]. Also history of recent surgery, a variable that has been detected to be associated with improved survival after 12 months, most probably separates patients with reversible causes of cardiocirculatory arrest postoperatively from those with progressive deterioration in terminal diseases. The total dose of adrenaline is known to adversely impact outcomes after CPR, and also in our study, survival rates decreased with increasing total adrenaline doses, along with prolonged CPR duration [24]. Age was found to be a factor of influence on ROSC but not on survival after 24 hours, to discharge, and after 12 months. In the literature, the prognostic value of age on survival after in-hospital CPR is discussed controversially [3]. In a study on in-hospital CPR in patients of 65 years and older, survival to discharge was reported to be 18.3% [7]. In our population, survival of patients older than 65 years 32.9% (32/110) to discharge and 13.6% (15/110) after one year.

Differences in event characteristics and survival outcomes depending on the time of the event, as previously described, were not observed in our study [6, 25, 26]. However, the number of patients may have been too small to detect specific effects.

Important are the results for the variables “witnessed arrest” and “CPR before arrival of the cardiac arrest team,” since these represent the only factors in our models that can be influenced effectively in the hospital prearrest setting. The parameter “witnessed arrest” was associated with an improved long-term survival rate not only 24 hours, but also for 12 months in multivariate analysis.

However, the results of the multivariate analyses have to be regarded with caution due to relatively small patient numbers and possible effects of multiple testing. Another limitation of our study is the lack of data on neurological outcomes. Neurological outcomes are part of the core data according to the Utstein-Style [27]. The percentage of patients with persistent disability is not known for our patients; therefore, also the interpretation of the survival rates should be regarded with caution.

Furthermore limiting to our results is missing data due to incomplete documentation by the cardiac arrest team at emergency setting, including medical data, for example, pupil findings [28]. Specific time intervals for the individual events, particularly the interval between the alarm and the arrival of the emergency team, were also not recorded. Minimizing the interval from event to CPR is crucial to impact survival [29], and the time to first defibrillation has been shown to be critical for survival [30]. Based on the floor plan of the hospital, the structure of the alarm system and random samples it is, however, estimated that the time from call to arrival is approximately 90 to 120 seconds on average and hereby is in an acceptable range. The cardiac arrest team was obliged to conduct CPR according to the respective active guidelines; nevertheless, no details on the technical conduction of CPR were recorded [31]. The total number of in-hospital CPR events in the respective period can be estimated to exceed the reported number, since in some intensive care units, medical emergencies are primarily handled by medical personnel on site, and the assistance of the hospital cardiac arrest team is only occasionally needed. Also, a small number of CPR events performed in general wards without the assistance of the hospital cardiac arrest team has to be assumed. Although the main medical diagnosis for the respective hospitalization is documented rigorously for each case, it can be speculated that secondary diagnoses may have been missed; therefore, comorbidities could not be tested for their relation with survival. Among comorbidities, sepsis, cancer, renal failure, and home-bound lifestyle have previously been described to be significantly associated with poor survival [3].

A remarkable and unexpected finding was the high percentage of medical emergencies not due to cardiac arrest. Over the nine-year period, the rate of alarm calls related to cardiac arrest decreased from 44% (for 1995 to 1997) to 34% (for 2004–2006). The four most frequent categories were airway problems, neurological emergencies, collapse and cardiac emergencies other than cardiac arrest. Interestingly, most of these symptoms are relevant in the context of the concept of medical emergency teams. We speculate that the assistance of the cardiac arrest team in the above-mentioned emergency conditions has helped to prevent cardiac arrest in a number of patients. Therefore, we conclude that the task profile of our cardiac arrest team increasingly exceeds the tasks of a traditional cardiac arrest team, it should rather be classified as an in-hospital emergency response system for a wide range of different medical emergency situations, not limited to cardiac arrest.

## 5. Conclusions

In conclusion, the observed survival rates after cardiopulmonary resuscitation by the cardiac arrest team at our center are in a notably high range compared to the literature. The variables predictive for survival are comparable to published data. However, with an increasing rate alarms not corresponding to cardiac arrests, the responsibilities and tasks of the cardiac arrest team in our institution exceed the provision of CPR, demanding additional competence and skills in the treatment of various in-hospital emergencies. To further improve in-hospital emergency response systems, we propose putting further emphasis on continuous training of all hospital members in immediate life support to provide cardiopulmonary resuscitation before arrival of the cardiac arrest team, and second, to include basic emergency response to a variety of medical conditions in the training of cardiac arrest team members.

## Figures and Tables

**Table 1:** Survival after CPR.

	All patients with CPR	ROSC	Survival to 24 hours	Survival to discharge	12-months survival^a^
	**189**	**124 (65.6%)**	**97 (51.3%)**	**57 (30.2%) **	**35 (18.5%)** ^ a^
Age in years	65.2 (±16.1)	63.4 (±17.9)	62.6 (±19.1)	62.0 (±21.2)	57.9 (±24.2)
>65 years	110	67	53	32	15

Male	132	91	74	42	24
Female	57	33	23	15	11

Witnessed arrest	99	71	59	37	26
Unwitnessed arrest	90	53	38	20	9

CPR before team arrival	102	74	64	37	22
No CPR before team arrival	87	50	33	20	13

CPR duration in minutes	20.4 (±17.7)	13.1 (±11.7)	12.2 (±12.2)	10.9 (±12.9)	9.8 (±14.6)
≤15 minutes	88	83	71	45	29
>15 minutes	101	41	26	12	6

Initial rhythm shockable^b^	61	44	40	30	18
Initial rhythm not shockable	126	80	57	27	17

Number of defibrillations^c^					
0	111	72	51	25	15
1–4	59	45	40	27	17
5–10	13	5	4	4	2
>10	5	1	1	0	0

Adrenaline total dose^d^					
0–4 mg	107	88	77	48	31
5–9 mg	28	15	8	4	2
10–14 mg	19	7	4	2	0
≥15 mg	16	4	1	0	0

Use of vasopressin for CPR^e^	49	21	13	4	2
Vasopressin not used	139	102	83	52	32

Arrest 8:00–19:59^g^	96	66	50	28	16
Arrest 20:00–7:59	89	54	43	25	16

Presumed cardiac etiology^f^	145	100	81	48	30
Presumed extracardiac etiology	42	22	14	7	3

History of malignancy	57	38	32	15	6
No history of malignancy	132	86	65	42	29

Recent history of surgery	28	24	22	17	12
No recent history of surgery	161	100	75	41	23

Location^h^					
General ward	134	89	69	36	18
Intensive Care Unit/intermediate care unit	12	9	7	6	6
Emergency ward	17	9	8	6	4
Cardiac catheterization lab	12	9	8	7	6
Other	13	7	5	2	1

^a^Age and CPR duration are expressed as mean and standard deviation. ^a^Missing data for seven patients; the survival rate was calculated assuming nonsurvival of these patients.

For the respective variables, data are missing ^b^for 2 patients, ^c^for 1 patient, ^d^for 19 patients, ^e^for 1 patient, ^f^for 4 patients, ^g^for 2 patients, and ^h^for 1 patient. “Initial rhythm shockable” denotes ventricular fibrillation or ventricular tachycardia; “initial rhythm not shockable” denotes either asystolia, pulseless electric activity, bradycardia, pacemaker, normal sinus rhythm, or other rhythm.

“Presumed cardiac etiology” is classified as either myocardial infarction, arrhythmia, or other cardiac etiology as the suspected first diagnosis for the cause of arrest.

“History of malignancy” denotes the diagnosis of any malignant tumor or disease during the respective hospitalization any time before cardiopulmonary resuscitation.

“Recent history of surgery” denotes a surgical procedure during the respective hospitalization during a maximum of 28 days before cardiopulmonary resuscitation.

**Table 2:** Multivariable logistic regression models for survival.

	ROSC	Survival to 24 hours	Survival to hospital discharge	12-month survival
Adrenaline total dose	**OR 0.89** (0.83–0.97) *P* = 0.006	**OR 0.81** (0.73–0.90) *P* < 0.001		**OR 0.56** (0.36–0.85) *P* = 0.007

Age	**OR 0.95** (0.90–0.99) *P* = 0.026	**OR 0.96** (0.93–0.99) *P* = 0.015		**OR 0.97** (0.94–1.00) *P* = 0.076

Duration of CPR	**OR 0.86** (0.81–0.92) *P* < 0.001	**OR 0.92** (0.88–0.95) *P* < 0.001	**OR 0.90** (0.82–0.99) *P* = 0.022	**0.93** (0.87–1.00) *P* = 0.001

Witnessed arrest		**OR 2.56** (1.01–6.50) *P* = 0.048		**3.34** (1.05–10.65) *P* = 0.041

Presumed cardiac etiology		**OR 4.89** (1.68–14.26) *P* = 0.004		**4.24** (0.88–20.41) *P* = 0.072

Initial rhythm shockable			**OR 13.41** (1.69–106.22) *P* = 0.014	

Recent history of surgery				**15.42** (3.02–78.79) *P* = 0.001

For ROSC, 80.6% of all cases were correctly classified with this model. The area under the ROC (receiver-operating characteristic) curve was 0.92 (SE 0.03 and 95% CI 0.85 to 0.96). For survival to 24 hours, 80.5% of all cases were correctly classified with this model (area under the ROC curve 0.91, SE 0.02, and 95% CI 0.85 to 0.95). For survival to hospital discharge, 85.7% of all cases were correctly classified with this model (area under the ROC curve 0.84, SE 0.085, and 95% CI 0.85 to 0.95). For survival after one year, 89.9% of all cases were correctly classified with this model (area under the ROC curve 0.93, SE 0.085, and 95% CI 0.88 to 0.96).
